# Mining the Protein Data Bank to improve prediction of changes in protein-protein binding

**DOI:** 10.1371/journal.pone.0257614

**Published:** 2021-11-02

**Authors:** Samuel Coulbourn Flores, Athanasios Alexiou, Anastasios Glaros

**Affiliations:** 1 Department of Biochemistry and Biophysics, Stockholm University, Solna, Sweden; 2 Department of Computer Science and Biomedical Informatics, University of Thessaly, Volos, Greece; 3 Eukaryotic Single Cell Genomics Facility, Science For Life Laboratory, Stockholm, Sweden; Universita degli Studi di Roma Tor Vergata, ITALY

## Abstract

Predicting the effect of mutations on protein-protein interactions is important for relating structure to function, as well as for *in silico* affinity maturation. The effect of mutations on protein-protein binding energy (ΔΔG) can be predicted by a variety of atomic simulation methods involving full or limited flexibility, and explicit or implicit solvent. Methods which consider only limited flexibility are naturally more economical, and many of them are quite accurate, however results are dependent on the atomic coordinate set used. In this work we perform a sequence and structure based search of the Protein Data Bank to find additional coordinate sets and repeat the calculation on each. The method increases precision and Positive Predictive Value, and decreases Root Mean Square Error, compared to using single structures. Given the ongoing growth of near-redundant structures in the Protein Data Bank, our method will only increase in applicability and accuracy.

## 1 Introduction

In this work we are interested in predicting the change in protein-protein interaction (PPI) energy (ΔΔG) resulting from amino acid substitutions at the protein-protein interface. This quantity determines the change in protein-protein binding affinity and is thus important for understanding signaling, complex assembly, host-pathogen interaction, and other functions. As the accuracy of computational methods increases, hope grows that these will match the accuracy of experimental ΔΔG measurement, heralding a new age of in the development of biologics–proteins which have therapeutic and/or diagnostic utility [1]. It would also help design proteins for purification, catalysis, and other purposes.

### 1.1. Methods of computing change in protein-protein interaction energy (ΔΔG)

ΔΔG = ΔG_mutant_− ΔG_wild-type_. ΔG_wild-type_ is the free energy change upon binding in the wild-type complex, while ΔG_mutant_ is the same quantity for the mutant complex. The equilibrium constant widely used in pharmacology is computed as Kd = exp(ΔG/RT), where R is the universal gas constant.

Many computational methods exist to calculate ΔΔG_predicted_, an estimate of the (known or unknown) experimental value, ΔΔG_experimental_. Some methods use reduced representations [1, 2], while others include all atoms [3]. such methods compute the protein-protein binding enthalpy (including electrostatic and van der Waals interactions) using physical formulae, but differ in the way they estimate the effect of solvent and side-chain entropy. The most successful and widely-used methods use implicit solvent to estimate these latter terms, namely they compute the solvent-accessible surface area on an atomic basis, then combine this quantity with the atom type and empirically-adjusted weight factors [3–6]. In recent years such approaches have made relatively small gains in accuracy.

Many limited-flexibility, implicit-solvent methods including FoldX offer good accuracy and economy [3, 7, 8]. Multiple workers have found it is actually counterproductive to minimize the structure globally (e.g. by Molecular Dynamics or MD) [9, 10], especially when the mutation induces small conformational changes [11]. Rather it is better to model the substitution and limit further modifications to those required for annealing the resulting steric clashes in a mostly-local minimization–in other words, a perturbative approach [3]. All force fields are inevitably biased by the data they are fitted to, and most are classically formulated, considering quantum mechanical effects only indirectly. Thus modeling can only reduce the accuracy of 3D atomic coordinates, compared to X-ray crystallography or other high-resolution experimental methods. Also, potentials which successfully predict changes in PPI energy upon mutation (ΔΔG) are typically trained on experimental structures [3]. All of this argues in favor of limited, local minimization. The downside of *local* minimization is that it makes the results dependent on the idiosyncrasies of the experimental coordinates which may reflect crystallization conditions, which would change upon mutation, or which represent only one of many thermodynamically accessible configurations. This work addresses this limitation of local minimization, by identifying and using additional structural data. These additional structures are rapidly increasing in number, as we will explain. We repeat the ΔΔG calculation over all such available structures, and average over the results to increase precision.

### 1.2. Near-redundant structures in the Protein Data Bank (PDB)

Although the era of fold discovery is over, the growth of structural data is still accelerating (S1 Fig). Many of the new structures differ only slightly from existing entries, having been obtained to e.g. seek higher resolution, determine the effect of a mutation, or add a ligand or subunit. In this work “near-redundant structures” refer to those which have the same (or nearly the same) composition, at least in the protein-protein interface of interest, but which were resolved in separate experiments or crystallographic units. The experimental effort is dramatically reduced when following proven protocols as opposed to solving proteins of previously unknown structure. Thus it is economical to solve the same complex to probe structural variations, improve resolution, etc. As an illustrative example, the complex of the human Growth Hormone (hGH) bound to two copies of its Receptor (hGHR) was resolved in [12]. There exists a mutant of hGH which binds one copy of hGHR, and the same work also reports the 1:1 complex structure [12]. The same lab then remodeled part of the interface by point and phage display mutagenesis and reported the structure [13]. Lastly, they solved the 1:1 complex again at improved resolution [14]. Even minor differences in biopolymer sequence, number of additional subunits, experimental conditions, and fitting procedure can be expected to introduce differences in atomic positions on the order of tenths of Ångströms. While for some purposes these differences may be insignificant, they lead to variations in predicted ΔΔG when using FoldX and other limited-flexibility methods.

### 1.3. Why does averaging improve precision?

Accuracy is the closeness of the prediction or measurement to the correct value, whereas precision refers to the closeness of independent predictions or measurements to each other. The principle underlying homologyScanner is that ZEMu and other methods which perform only a local minimization are subject not only to limitations in accuracy (due to biases in the ΔΔG prediction potential, and to errors in the ΔΔG_experimental_ used as a gold standard) but also in precision (due to particularities of the coordinate sets used).

According to the central limit theorem, for N independent random variables distributed with mean μ and standard deviation σ, the sample mean approaches a normal distribution with mean μ and standard deviation $\sigma /\sqrt{N}$. Here the ΔΔG_predicted_ computed using a single structure would be a random variable sampled from an underlying normal distribution of unknown mean μ and standard deviation *σ*. A ΔΔG_predicted_ averaged from *multiple* calculations would have a smaller standard deviation, $\sigma /\sqrt{N}$, about the same mean μ.

Thus averaging multiple calculations obtained using independent structures provides a more-accurate estimate of the underlying mean μ. Note that even in the hypothetical case of very large N, μ would still be the mean of many individual values of ΔΔG_predicted_, subject to biases in the FoldX potential, and may not converge to an accurately measured ΔΔG_experimental_. Also the latter number depends on experimental conditions. And so though precision is increased by our method, one must consider limitations in the force fields and experimental measurements.

## 2 Methods

**Fig 1:**
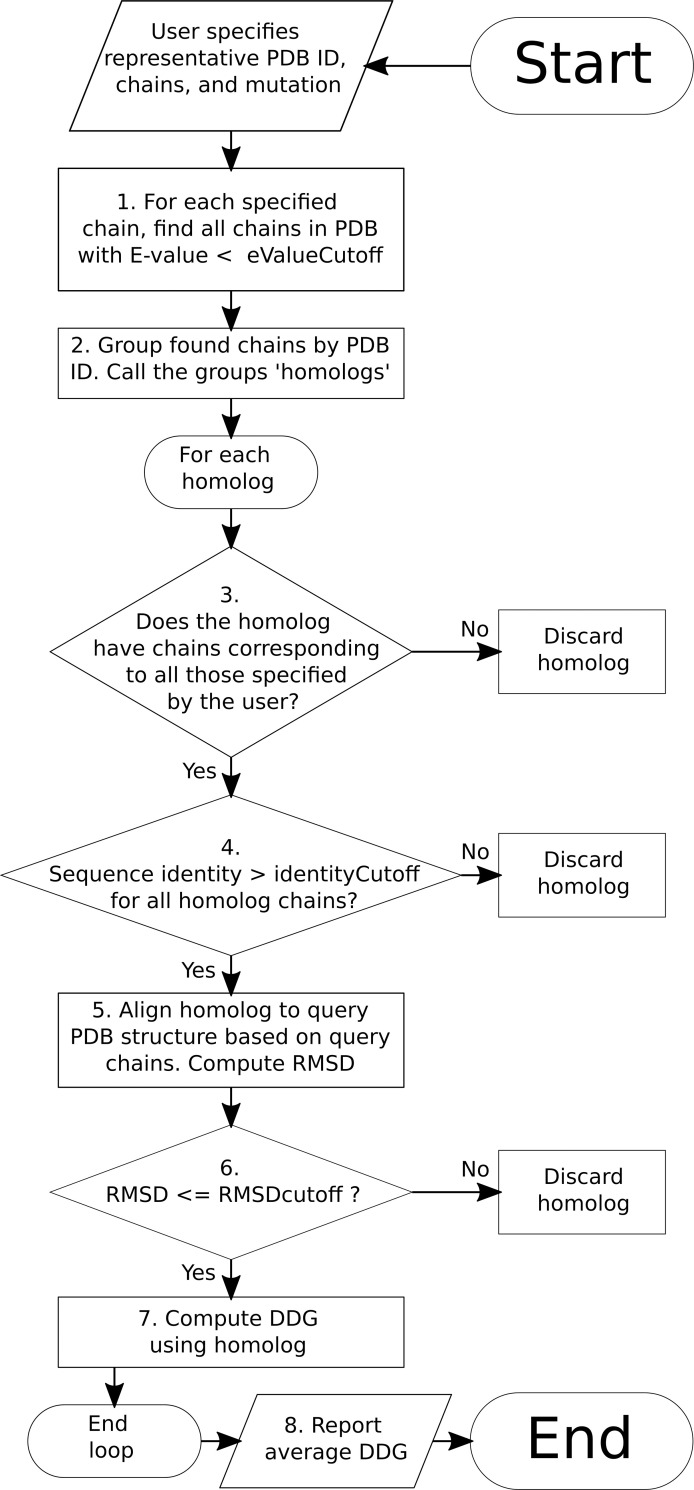
Program flow. The user must provide an initial Protein Data Bank (PDB) ID, specify which relevant chains are in which of two interacting complexes (irrelevant chains may be left out). 1. The fasta_lwp program searches the PDB for structures containing chains homologous (E-value below eValueCutoff, here 10^−11^) to those specified by the user. 2. We group the thus-discovered homolog chains by PDB ID, each such PDB ID is referred to as a “homolog.” We loop over the homologs, performing three checks on each. 3. As a first check, we determine whether the thus-discovered homologs contain chains corresponding to all those specified by the user; those not having all such chains are discarded. 4. Homologs in which all chains do not have at least 90% sequence identity vs. the corresponding user-specified chain are discarded. 5. We perform a rigid alignment of the entire homolog against the user-specified structure, based only on the user-specified chains. Non-corresponding (extraneous) chains are moved along with the rest of the complex. This is the most computationally-expensive process, but only needs to be done once for homolog that makes it to this step; results of all three checks are saved persistently. 6. If RMSD > 6.0 Å (again based on corresponding chains), we discard the homolog. Most homologs which are rejected at this step contain the correct chains but in a different configuration. 7. We then compute the ΔΔG for the user-requested mutation, using the homolog structure and FoldX4. Steps 3–7 are repeated for each homolog. 8. We average ΔΔG over all homologs that reached and completed step 7 and report the result.

In our method, the user proposes a mutation, the PDB identifier of a single”query” structure, and two lists of chains, one for each subunit in the interaction of interest. Lastly, the user specifies the chain ID, residue position, and substituted residue type, for one or multiple simultaneous substitutions. The user typically needs not perform any further actions until the calculation completes, the remaining steps are automated, per the flowchart (Fig 1).

The automated steps are, in short: 1) Do a sequence search of the PDB for structures having all the chains specified by the user, within a specified e-value and sequence identity. 2) Do a structural alignment between the query and each subject structure, and accept the subject if the RMS Deviation (RMSD) meets the cutoff. 3) Translate the mutation to the numbering system of the subject structure, and compute ΔΔG. 4) Average ΔΔG over all structures, and report to the user.

**Fig 2:**
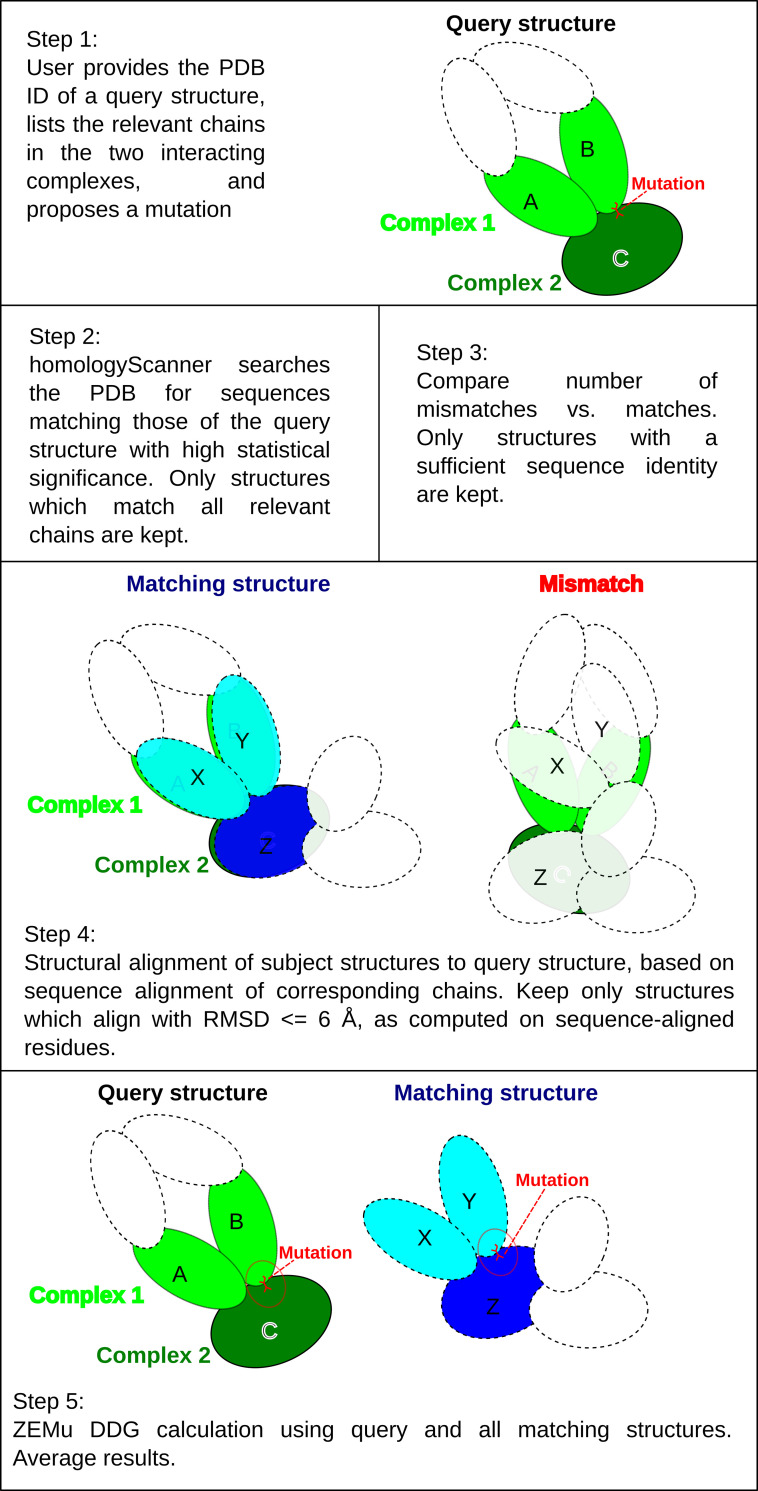
Illustration of the sequence and structure matching procedure.

The procedure is illustrated graphically in Fig 2. homologyScanner is an extension of MacroMoleculeBuilder (MMB), written in C++ and made available on github.

### 2.1. MacroMoleculeBuilder (MMB)

MMB is an open-source, general-purpose, multiscale modeling code. Its internal-coordinate framework [15] gives us full control over the flexibility of our molecular system, thus one can have chains which are fully rigid, fully flexible, or which are rigid in some parts and flexible in others. In past work we have used MMB for applications as diverse as morphing [16], local minimization [17], and fitting to low-resolution electron density maps [18]. MMB can also do homology modeling, which may be considered as alignment of a flexible chain (of presumed unknown structure) to a rigid chain of known structure [19]. In a related technique, both chains can be partially-flexible or fully-rigid, and here the alignment can be called template docking [20] or simply rigid alignment, depending on how constraints are applied. MMB can also use the Kabsch algorithm to compute the minimum Root Mean Square Deviation (RMSD) with which two complexes can be aligned–this is a very fast operation.

### 2.2. Sequence search and alignment

homologyScanner starts by searching the PDB [21] for chains which match the sequence of the user-specified chains with very high statistical significance (e-value < = 10^−11^). Only structures which contain all the user-specified chains, each satisfying the e-value requirement, are kept and the rest are discarded. homologyScanner then uses the SeqAn-based [22] alignment tools in MacroMoleculeBuilder (MMB) to compute the sequence identity for each corresponding chain, viz:

Matching residues / minimum(query chain length, subject chain length)

Structures which do not satisfy the minimum sequence identity (> 90%) are discarded. We thus know that the remaining structures have the relevant chains, but do not yet know whether they have the correct tertiary and quaternary structure. For that we perform the final structural check using MMB as follows.

### 2.3. Structural alignment

The Kabsch structural alignment is based on residue-residue (and ultimately atom-atom) correspondence between the query and subject structures, which we obtain from the mentioned sequence alignment. MMB can robustly deal with missing or non-canonical atoms (often encountered in PDB structures), usually without user intervention [16]. The Kabsch alignment gives us the RMSD of the query vs. subject complex. If the RMSD meets a cutoff threshold (< = 6Å), the homolog complex is then passed on to the ΔΔG calculation. Note that this differs from the procedure of [23], in which the PDB is searched on structure but not sequence; we wished to use only structures of very similar sequence to maintain accuracy.

### 2.4. ΔΔG calculation

The user specifies a mutation(s) with chain ID(s) and residue number(s) in the context of the query structure. However different subject structures may employ different residue numbering conventions. We translate the user-specified mutation into the numbering system of the subject structure on the basis of the sequence alignment. We compute ΔΔG_predicted_ on the query and all subject structures, using FoldX. FoldX was originally formulated and trained to predict changes in stability rather than PPI energy. However in [17] and in this work, we show that it is also highly effective for the latter. SKEMPI was compiled long after [3], and so overfitting is not a significant problem. Readers are referred to [3] for details on PPI energy evaluation in FoldX.

## 3 Results

We benchmarked homologyScanner on the dataset used in [17], comprising 1243 mutations (see Table 1, dataset A). This is a very diverse dataset of 1243 mutants, including some mutants with single-substitutions and some with multiple simultaneous substitutions. We first tried using only single structures, as done in [17], and then repeated using multiple structures and quantified the improvement in correlation and Root Mean Square Error (RMSE) [17]:

$$RMSE=\sqrt{\frac{\sum_{i=1}^{N}{\left({\Delta \Delta G}_{i,predicted}-{\Delta \Delta G}_{i,experimental}\right)}^{2}}{N}}$$

We also tested a subset of the above, comprising single- and multiple-substitution mutants for which multiple structures were available (Table 1, dataset B). We further subdivided into single- and multiple-substitution mutants available (Table 1, datasets C and D). The RMSE decreases as number of available homologs increases from 1 to 4 (S2 Fig). However from 5 homologs onwards RMSE increases as number of data points becomes small and begins to consist of ΔΔG_experimental_ measurements from a single lab.

**Table 1:** Comparison of computing ΔΔG using single vs. multiple structures, for several subsets of our benchmark set.

	Dataset A		Dataset B		Dataset C		Dataset D	
Simultaneous substitutions	Single or multiple		Single or multiple		Single		Multiple	
Structures available	Single or multiple		Multiple		Multiple		Multiple	
Structures used	Single	Single or multiple	Single	Multiple	Single	Multiple	Single	Multiple
N	*1190*	*1190*	*725*	*725*	*522*	*522*	*203*	*203*
RMSE (kcal/mol)	*1*.49*	*1*.*41*	*1*.54*	*1*.*37*	*1*.19*	*1*.*10*	*2*.17*	*1*.*96*
Correlation	.61*	*0*.*65*	*0*.59*	*0*.*65*	*0*.51*	*0*.*56*	*0*.56*	*0*.*67*
p-value	*4E-5*		*3E-6*		*1E-4*		*8E-4*	

Row label *Simultaneous substitutions*: how many simultaneous substitutions in each mutant? Can be single-substitutions, multiple-substitutions, or a mixed set. Row label *Structures available*: how many structures are available for a given mutation in the dataset? For dataset in column A, used in [17], multiple structures are available for but not all mutations, the remaining three datasets comprise only mutants for which multiple structures are available. Row label *Structures used*: How many of the available structures were used? For each dataset we compare use of all available structures (homologyScanner) vs. use of only one structure. Bottom: N, number of mutants in the dataset; RMSE, Root Mean Square Error; Correlation. Note that in all cases use of multiple structures yields lower RMSE and higher (or equal) correlation than use of single structures. Best results are with single-substitution mutants (as found in [17]), for which multiple structures are available (dataset **A**). p-values give the probability that the difference in performance could have been observed by chance, and were computed using the Wilcoxon signed-rank test, comparing the squared-errors when using single vs. multiple structures.

*Where multiple structures were available and only one was used, choice was made at random, and RMSE and correlation averaged over five randomizations.

**Fig 3:**
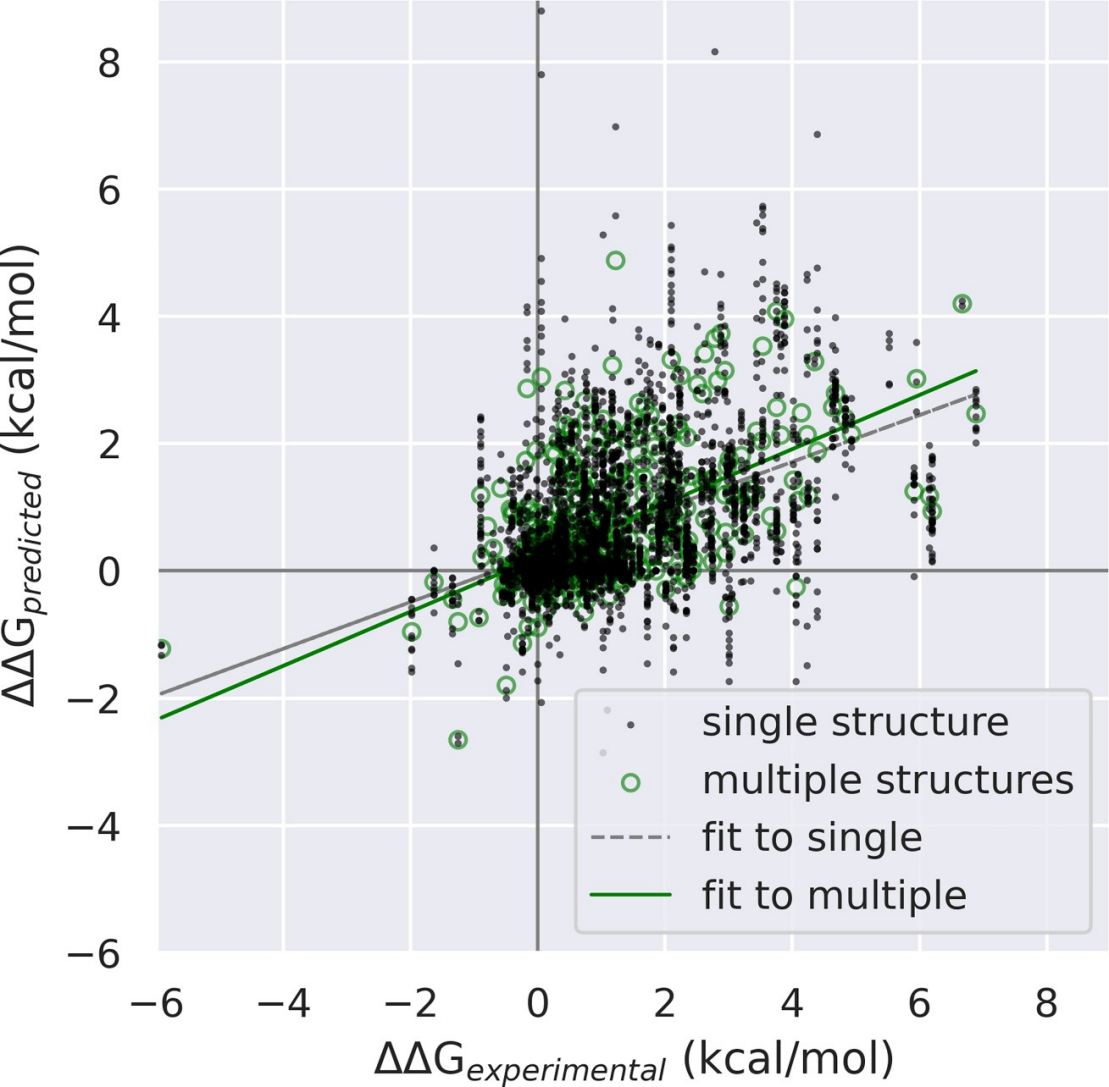
Scatterplot of ΔΔG_predicted_ vs. ΔΔG_experimental_, for dataset C (single-position substitutions, where more than one structure was available). Green circles: mutants averaged over multiple structures, N = 522. Black dots: mutants computed on a single structure—as multiple structures were available for each mutant, this has a higher N = 4028. Note the clear outliers are all single-structure points. Note the third quadrant is populated with True Positives— ΔΔG_predicted_ and ΔΔG_experimental_ both negative. On the other hand, the fourth quadrant, representing False Positives, does not have any multiple-structure results below ΔΔG_predicted_ < -0.65 kcal/mol. The improvement in Positive Predictive Value is discussed elsewhere in this work.

A scatterplot (Fig 3) of ΔΔG_predicted_ vs. ΔΔG_experimental_ for dataset C, shows the effect of averaging on outliers.

**Fig 4:**
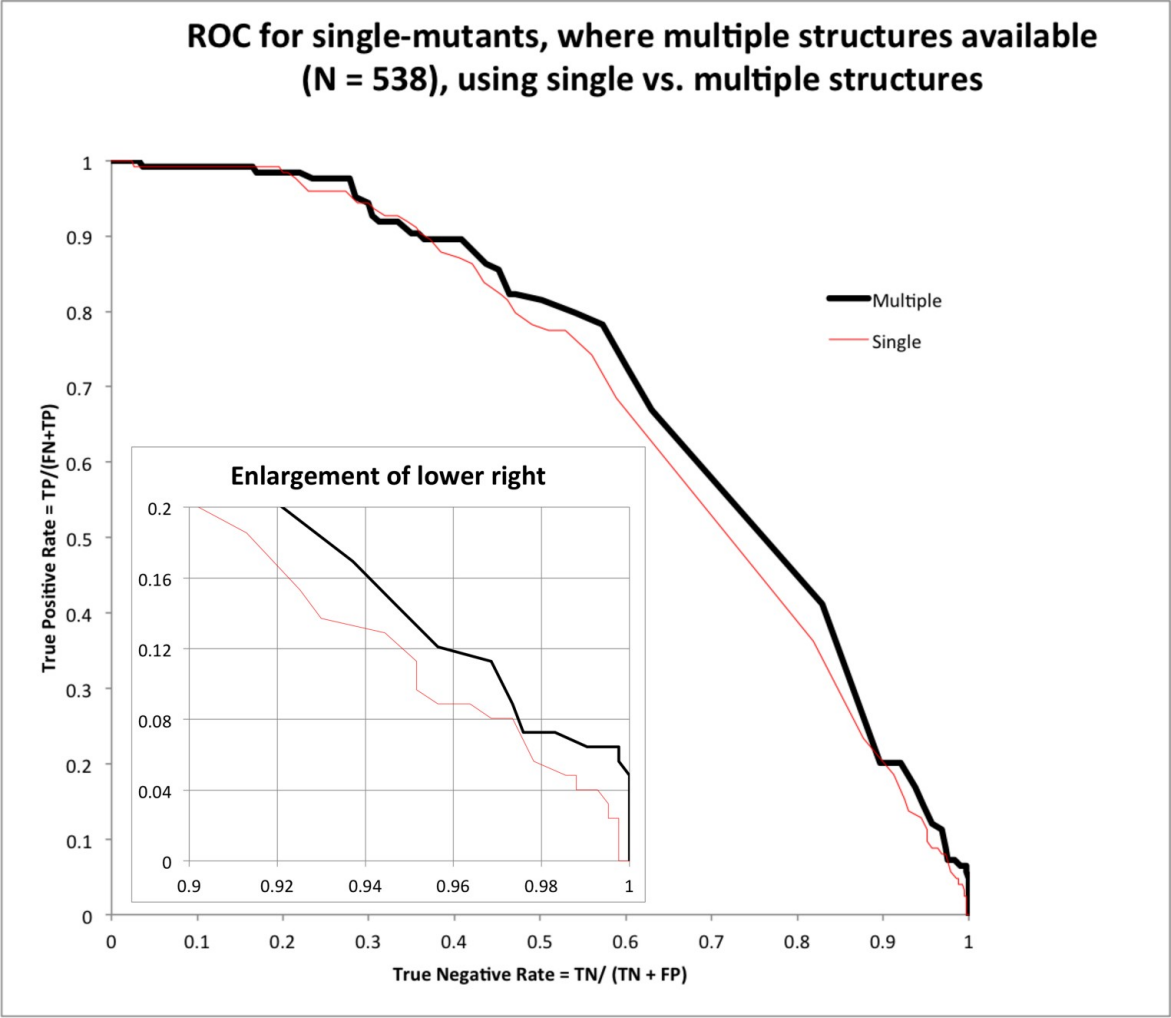
Receiver Operating Characteristic, comparing homologyScanner vs. calculation on single structures. Here the Test Positives are defined as mutants with ΔΔG_predicted_ < threshold, where the threshold is varied.

We also computed a Receiver Operating Characteristic (ROC) curve (Fig 4). This plots the True Positive Rate (TPR) vs. True Negative Rate (TNR) as the ΔΔG_predicted_ *threshold* is moved from ΔΔG_predicted_ = +∞ (loosest) to -∞ (strictest). Mutations with ΔΔG_predicted_ < *threshold* are taken to be test positives, mutations with ΔΔG_experimental_ < 0 are taken to be Gold Standard positives, and so e.g. mutations in the intersect set (i.e. those that meet both of these criteria) are True Positives (TP). Accordingly, FP (False Positives) are those mutations for which with ΔΔG_predicted_ < *threshold* but ΔΔG_experimental_ ≥ 0. The rest of the quantities (TN: True Negatives, FN: False Negatives, etc.) are computed accordingly. The ROC was also computed based on single structures, and on multiple structures for comparison.

**Fig 5:**
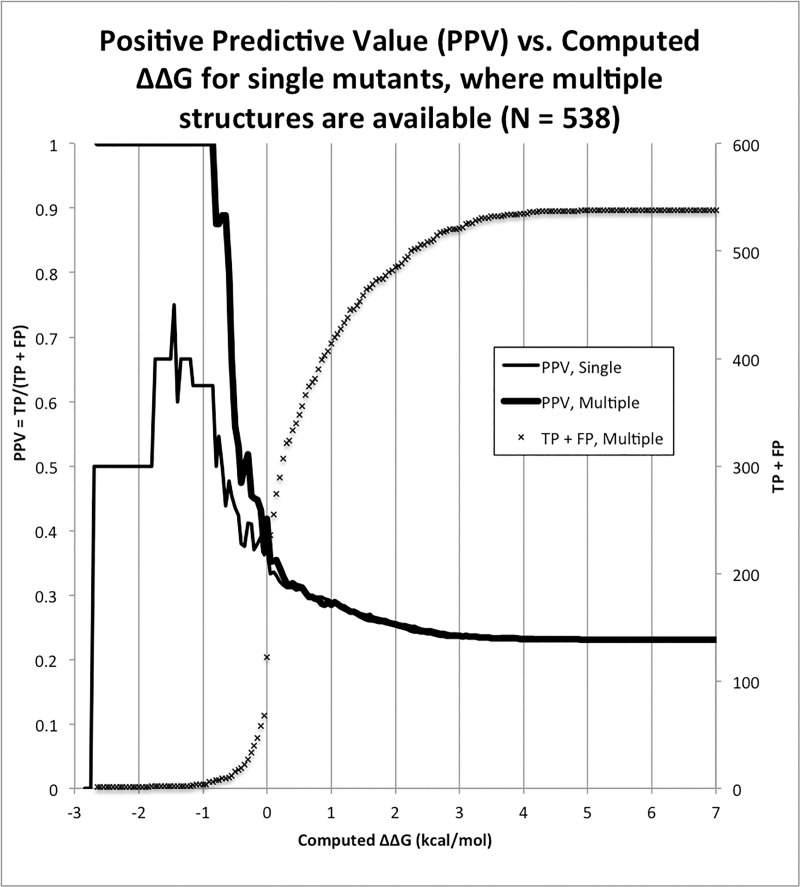
Positive Predictive Value (PPV) for single vs. multiple structures. TP + FP is the denominator of PPV, so we emphasize that this quantity becomes small for ΔΔG_predicted_ < -1 kcal/mol (crosses). This is why the PPV becomes erratic, at least for single structures.

Another useful quantity is the Positive Predictive Value (PPV) = TP/(TP + FP). This answers the question: for a given threshold, what fraction of test positives will be TPs? If the goal is to get ΔΔG > 0, then PPV tells us which fraction would have achieved this, for a given test threshold. Again we compute for single as well as multiple structures for the full range of test thresholds (Fig 5).

## 4 Discussion

As noted previously many ΔΔG prediction methods that limit structural rearrangements, particularly in regions distant from the mutation site, can yield good results, compared to no minimization or minimization of entire structures [3]. However a local minimization leaves us vulnerable to structural idiosyncrasies of the structure employed. In the present work, we diversify the coordinate data by identifying additional complexes including the relevant chains, in the relevant quaternary arrangement. Significantly, we introduce no adjustable parameters and so our results should apply to other potentials and localized minimization methods. The part of the error due to experimental uncertainty and limitations of the DDG potential, remains unchanged in our work but is the topic of ongoing research in the field [3, 9, 24].

Our benchmarking was done using the dataset of [17], itself compiled from SKEMPI [25]. The full dataset **A** contains mutants with single and multiple simultaneous substitutions. More to the point, for some mutants in dataset A only one structure is available, whereas for others there are several similar structures available (in one case 18 were found, see S1 Table). When only one structure is available of course homologyScanner makes no improvement, but since for many there were multiple structures, homologyScanner reduced RMSE by about 0.1 kcal/mol.

The more relevant comparison is the case for which multiple structures are available (datasets B, C, and D). Dataset C comprises only single-substitution, D comprises multiple-substitution mutations, while B contains both. homologyScanner presented the largest RMSE improvement for D, but error was high overall, so in general we do not recommend using homologyScanner for multiple-substitutions. For the single-substitutions (dataset C), the best RMSE of all, 1.04 kcal/mol, was obtained, better than reported in related work [3, 17].

Aggregate results however are only part of the story. Fig 3 highlights another important feature of homologyScanner:F whereas several outliers are evident when using single structures, there are arguably *zero* outliers when using multiple structures. For a given ΔΔG_experimental_, ΔΔG_predicted_ is consistently closer to the trendline for multiple than for single structures. One may note that the slope ΔΔG_predicted_/ΔΔG_experimental_ is not unity, this is a characteristic of FoldX which we do not reparameterize here; in any case the slope itself is not as important as the statistical measures of accuracy.

ROC curves are commonly used to evaluate binary classifiers with an adjustable threshold–in this case, we can classify mutations into those predicted to decrease ΔΔG (improve affinity), vs. those that should increase ΔΔG or leave it neutral. In ROC curves, Area Under the Curve (AUC) and slope at the point TNR = 1, TPR = 0 are two important measures. Larger AUC’s correspond to more significant classifiers, whereas steeper slope indicates better performance for the highest-confidence cases, here those with lowest computed ΔΔG. Both quantities are larger when using multiple structures (Fig 4).

But perhaps the most important statistic, again for the purposes of design, is PPV, plotted in Fig 5. For single structures, PPV fluctuates around 0.5 for the highest-confidence mutants, that is to say in the range of ΔΔG_predicted_ < -1 kcal/mol. For multiple structures, in contrast, PPV is a solid 1.0 in the same range of ΔΔG_predicted_. To reiterate, in an experiment *all* such mutants would have been found to improve affinity. While we believe this result is important and impressive, we also urge the reader to be cautious. This dataset is compiled from *published* data, which we strongly suspect contains more affinity-improving mutations than would be obtained by random mutagenesis. We reason that may investigators are looking to improve affinity, and will use tools at their disposal–published and unpublished data, structural calculations, bioinformatics, etc., prior to attempting a new mutation–and if they do not succeed they may decide not to publish. So while the case is strong for using multiple structures, we believe PPV will be less than unity in new applications.

In conclusion, we have presented a protocol for taking advantage of the growing accumulation of near-redundant structures in the PDB to improve prediction of ΔΔG. Though the approach is simple, it provides an improvement which is remarkable since clearly demonstrable improvements in ΔΔG accuracy have been slow in recent years. The method should be compatible with other ΔΔG prediction methods which use perturbative energy minimizers, in addition to FoldX. As multiple programs are required to implement homologyScanner efficiently, and since there is considerable incentive to reuse calculations, we make the method publicly available on an easy to use web server.

## 5 Distribution

**Fig 6:**
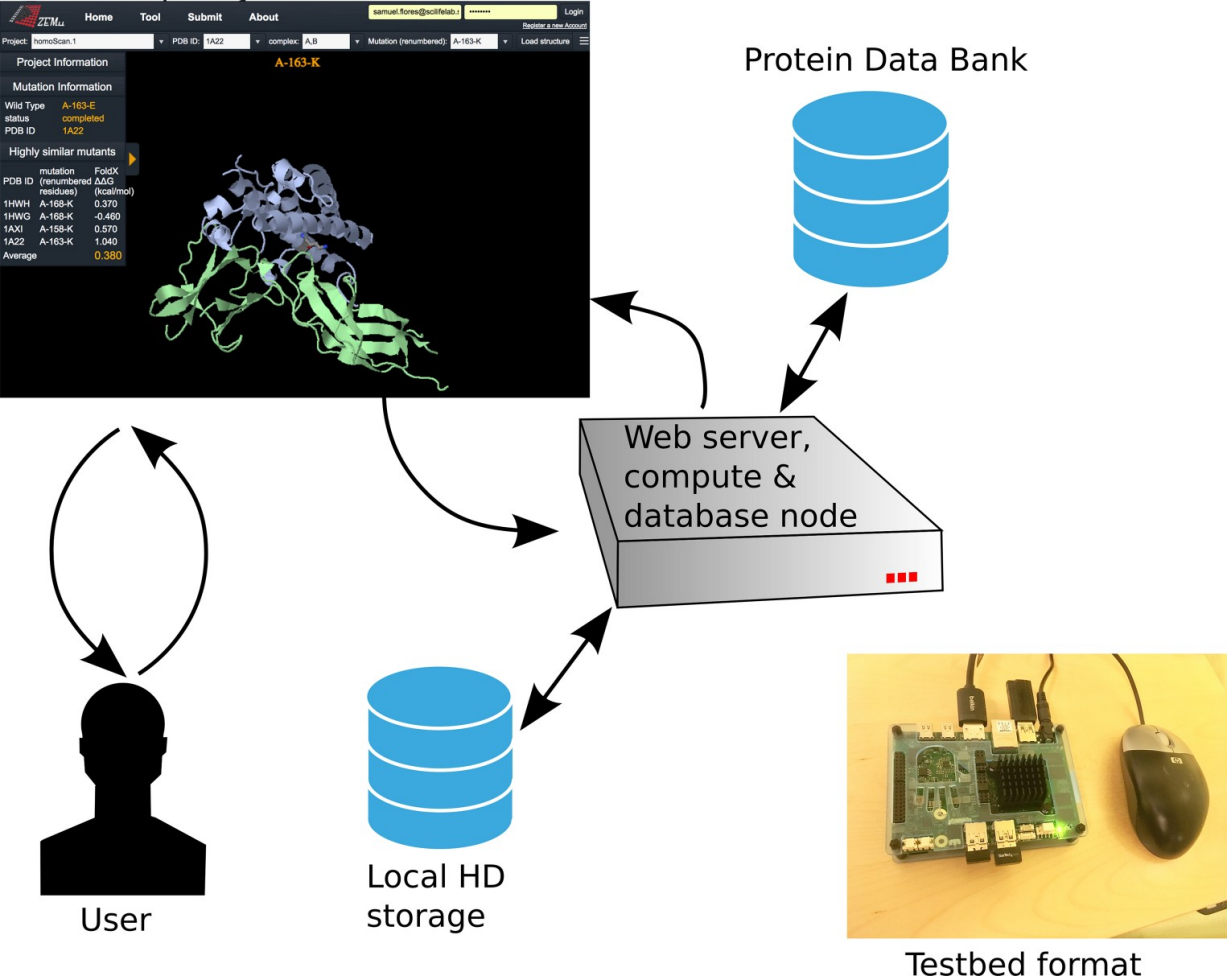
The homologyScanner public web server. Users can provide PDB ID, chose chains in each of two subunits, and specify a mutation to be computed. FoldX ΔΔG is computed for the query and all matching complexes and reported to the user. The results are available for browsing by others. Compute nodes are needed only for high-throughput runs. The software components are available on github, simtk.org, and dockerhub. A server has also been set up on a single-board computer for private deployment.

HomologyScanner is available on a public server at biodesign.scilifelab.se. The setup is shown in Fig 6. To request a ΔΔG calculation, the user goes to the *Submit* tab and provides the PDB ID of one suitable structure, and specifies the relevant chains in subunit 1 and subunit 2 of the interaction of interest (chains not in the interface can be left out). The user then specifies the mutation to be computed (one to four simultaneous substitutions). homologyScanner is then invoked, meaning the PDB is searched for structurally similar complexes, the FoldX ΔΔG calculation is performed for all such complexes found, and the user is notified by email (usually within a few hours, depending on queue status and job characteristics) when the job is done. The server saves all results, so the structure search needs be done only once per PDB ID and definition of subunits 1 and 2, and each FoldX calculation is only done once in total.

There is also a *View* tab where the public can browse all results by selecting a PDB ID, subunits, and mutation (all from drop-down lists). They will then see the ΔΔG for all structural homologs, as well as the average ΔΔG. A Jsmol window displays the protein structure in *Cartoon* render style, with the mutation highlighted in *Sticks* style.

The web server itself comprises a computer with at least two CPU cores, one of which is responsible for running Apache, MySQL, and other web services. The remaining cores are managed by the SLURM queueing system. The web server submits homologyScanner jobs to this queue upon user request. These jobs call homologyScanner itself, which interacts with the PDB to perform the sequence and structure search. homologyScanner spawns one *breeder* job for each suitable homologous structure found. Breeder is a program introduced in [17] which manages FoldX and stores ΔΔG and related results in the MySQL database.

Privacy may be important for some users, for example academics with unpublished data, or product developers in the pharmaceutical industry. For such users we have prepared a low-cost Udoo X86 Ultra single board computer implementation. The compact (120x85 mm) and light (under 200 g, excluding power adapter) format means it can easily be posted to the an academic or industry user. The X86 architecture ensures ease of compilation and update, compared to ARM architectures used by other single-board computers. The computer has a 2.56 GHz Intel Pentium quad-core processor. One core is used for running the web server, including MySQL database, and 1–3 cores are managed by SLURM for running homologyScanner. The computer can be fitted with an M.2-format Solid State Drive with up to 1TB capacity (we used 512 GB) as well as external HDs. We used a StarTech 300Mbps mini-wireless network adapter. An HDMI and three USB-3.0 type A ports mean it can be connected to a display, keyboard, mouse, and wireless network adapter. Alternatively such users can provide their own hardware (with mysql, docker, slurm) and use the dockerhub image (samuelflores/mmb-ubuntu:homologyscanner) which contains MMB, Breeder, and homologyScanner.

DDGs averaged over multiple structures are provided in.csv format, as (in a separate file) are those computed using only single structures, at biodesign.scilifelab.se/publicdata.

## Supporting information

S1 Table Root Mean Square Error (RMSE) grouped by primary (author-provided) PDB ID, for dataset A (N = 1190). Number of homologs ranged from 1 (41 PDB IDS, accounting for a total of 465 mutants) to 29 (2PCC, 12 mutants). Some PDB IDs appear more than once, because not all calculations converged for all homolog structures. Note the particularly low RMSE for 1A22 and 1DAN, which dominate the statistics for number of homologs = 3 and 4. This may reflect a low error in the ΔΔG_experimental_, due to quality experimental work in the Wells and Edgington labs, on proteins of medical interest.
(DOCX)

S1 Fig Growth in Protein Data Bank depositions has been approximately quadratic since 1992. Depositions *per year* are growing approximately linearly (slope of 461 structures/year). Accordingly *total* structures are can be fitted to good approximation by 237*(year-1992)^**2**^–466*(year-1992) + 2523 (using numpy’s polyfit function). Interestingly, the largest increase was in 2020, despite (or perhaps because of) the Covid crisis [Acta Crystallogr D Struct Biol. 2020 Apr 1;76:311–312].
(TIF)

S2 FigRoot Mean Square Error (RMSE) decreases with number of structures used.All data points are over the same set of mutants, namely single- and multiple-substitutions mutants, where 4 or more structures are available, N = 511. For all mutants, we randomly selected n = 1, 2, 3, and 4 of the available structures and computed RMSE; we repeated this five times. A maximum of *n* = 4 was selected to include the high-quality data associated with 1A22, and also to have a sufficiently high N. We suggest a model (based on normally distributed errors) in which total error *σ_total_* is given by:

$$\sigma_{total}=\sqrt{\left(\frac{\sigma_{singlestruct}^{2}}{n}\right)+\sigma_{systematic}^{2}}$$

Thus for large *n*, the total error would converge to *σ_systematic_* – the error due to the perturbative assumption, crystallization artifacts, biases in the underlying force field, etc. The RMSE qualitatively appears to have this convergence, though the data admit other models.(TIF)

## References

[1] Dehouck Y, Kwasigroch JM, Rooman M, Gilis D. BeAtMuSiC: Prediction of changes in protein-protein binding affinity on mutations. Nucleic Acids Res. 2013;41: W333–9. doi: 10.1093/nar/gkt450 23723246 PMC3692068

[2] Dehouck Y, Grosfils A, Folch B, Gilis D, Bogaerts P, Rooman M. Fast and accurate predictions of protein stability changes upon mutations using statistical potentials and neural networks: PoPMuSiC-2.0. Bioinformatics. 2009;25: 2537–2543. Available: papers://c33b182f-cf88-47e8-a9c5-ad67b5626483/Paper/p1966 doi: 10.1093/bioinformatics/btp445 19654118

[3] Guerois R, Nielsen JE, Serrano L. Predicting changes in the stability of proteins and protein complexes: a study of more than 1000 mutations. J Mol Biol. 2002;320: 369–87. doi: 10.1016/S0022-2836(02)00442-4 12079393

[4] Cornell Cieplak P., Bayley C. I., Gould I. R., Merz K. M., Ferguson D. M., Spellmeyer D. C., et al. A second generation force field for the simulation of proteins, nucleic acids and organic molecules. J Am Chem Soc. 1995;117: 5179–5197.

[5] Massova I, Kollman PA. Combined molecular mechanical and continuum solvent approach (MM-PBSA/GBSA) to predict ligand binding. Perspect Drug Discov Des. 2000;18: 113–135. doi: 10.1023/A:1008763014207

[6] Li M, Simonetti FL, Goncearenco A, Panchenko AR. MutaBind estimates and interprets the effects of sequence variants on protein-protein interactions. Nucleic Acids Res. 2016;44: W494–W501. doi: 10.1093/nar/gkw374 27150810 PMC4987923

[7] Lowegard Anna U. AND Frenkel MSANDHGTANDJJDANDOAAANDDBR. Novel, provable algorithms for efficient ensemble-based computational protein design and their application to the redesign of the c-Raf-RBD:KRas protein-protein interface. PLOS Comput Biol. 2020;16: 1–27. doi: 10.1371/journal.pcbi.1007447 32511232 PMC7329130

[8] Pires DE V, Ascher DB, Blundell TL. mCSM: predicting the effects of mutations in proteins using graph-based signatures. Bioinformatics. 2014;30: 335–42. doi: 10.1093/bioinformatics/btt691 24281696 PMC3904523

[9] Petukh M, Li M, Alexov E. Predicting Binding Free Energy Change Caused by Point Mutations with Knowledge-Modified MM/PBSA Method. PLoS Comput Biol. 2015;11: e1004276. doi: 10.1371/journal.pcbi.1004276 26146996 PMC4492929

[10] Beard H, Cholleti A, Pearlman D, Sherman W, Loving KA. Applying physics-based scoring to calculate free energies of binding for single amino acid mutations in protein-protein complexes. PLoS One. 2013;8: e82849. doi: 10.1371/journal.pone.0082849 24340062 PMC3858304

[11] Kellogg E, Leaver-Fay A, Baker D. Role of conformational sampling in computing mutation-induced changes in protein structure and stability. Proteins Struct Funct Bioinforma. 2010;79: 830–838. Available: papers://c33b182f-cf88-47e8-a9c5-ad67b5626483/Paper/p2145 doi: 10.1002/prot.22921 21287615 PMC3760476

[12] Receptor I, Sundström M, Lundqvist T, Rödin J, Giebel LB, Milligan D, et al. Protein Chemistry and Structure: Crystal Structure of an Antagonist Mutant of Human Growth Hormone, G120R, in Resolution Crystal Structure of an Antagonist Mutant of Human Growth Hormone, G120R, in Complex with Its Receptor at 2. 9 Å Resolution *. 1996. doi: 10.1074/jbc.271.50.32197 8943276

[13] Atwell S, Ultsch M, De Vos AM, Wells JA. Structural Plasticity in a Remodeled Protein-Protein Interface. Science (80-). 1997;278. 10.1126/science.278.5340.1125 9353194

[14] Clackson T, Ultsch MH, Wells J a, de Vos a M. Structural and functional analysis of the 1:1 growth hormone:receptor complex reveals the molecular basis for receptor affinity. J Mol Biol. 1998;277: 1111–28. doi: 10.1006/jmbi.1998.1669 9571026

[15] Flores SC, Sherman MA, Bruns CM, Eastman P, Altman RB. Fast flexible modeling of RNA structure using internal coordinates. IEEE/ACM Trans Comput Biol Bioinforma. 2011;8. doi: 10.1109/TCBB.2010.104 21778523 PMC4428339

[16] Tek A, Korostelev AA, Flores SC. MMB-GUI: a fast morphing method demonstrates a possible ribosomal tRNA translocation trajectory. Nucleic Acids Res. 2016;44: 95–105. doi: 10.1093/nar/gkv1457 26673695 PMC4705676

[17] Dourado DFAR, Flores SC. A multiscale approach to predicting affinity changes in protein-protein interfaces. Proteins. 2014;82: 2681–90. doi: 10.1002/prot.24634 24975440

[18] Flores SC. Fast fitting to low resolution density maps: elucidating large-scale motions of the ribosome. Nucleic Acids Res. 2014;42: 1–10. doi: 10.1093/nar/gkt1324 24081579 PMC3902909

[19] Flores SC, Wan Y, Russell R, Altman RB. Predicting RNA structure by multiple template homology modeling. Pac Symp Biocomput. 2010; 216–27. Available: http://www.pubmedcentral.nih.gov/articlerender.fcgi?artid=2872935&tool=pmcentrez&rendertype=abstract doi: 10.1142/9789814295291_0024 19908374 PMC2872935

[20] Dourado DFAR Flores SC. Modeling and fitting protein-protein complexes to predict change of binding energy. Nat Sci Reports. 2016;6: 25406. doi: 10.1038/srep25406 27173910 PMC4865953

[21] Lopez R, Cowley A, Li W, McWilliam H. Using EMBL-EBI Services via Web Interface and Programmatically via Web Services. Curr Protoc Bioinforma. 2014;2014: 3.12.1–3.12.50. doi: 10.1002/0471250953.bi0312s48 25501941 PMC4312015

[22] Döring A, Weese D, Rausch T, Reinert K. SeqAn An efficient, generic C++ library for sequence analysis. BMC Bioinformatics. 2008;9: 11. doi: 10.1186/1471-2105-9-11 18184432 PMC2246154

[23] Sippl MJ. Boltzmann’s principle, knowledge-based mean fields and protein folding. An approach to the computational determination of protein structures. J Comput Aided Mol Des. 1993;7: 473–501. doi: 10.1007/BF02337562 8229096

[24] Brender JR, Zhang Y, Cargill M, Altshuler D, Ireland J, Sklar P, et al. Predicting the Effect of Mutations on Protein-Protein Binding Interactions through Structure-Based Interface Profiles. Jernigan RL, editor. PLOS Comput Biol. 2015;11: e1004494. doi: 10.1371/journal.pcbi.1004494 26506533 PMC4624718

[25] Moal IH, Fernández-Recio J. SKEMPI: a Structural Kinetic and Energetic database of Mutant Protein Interactions and its use in empirical models. Bioinformatics. 2012;28: 2600–7. doi: 10.1093/bioinformatics/bts489 22859501

