# Supplementary material for: Mining the Protein Data Bank to improve prediction of changes in protein-protein binding
Source: PLoS One. 2021 Nov 2;16(11):e0257614. doi: 10.1371/journal.pone.0257614 (PMC8562805; doi:10.1371/journal.pone.0257614)
Supplement: S1 Table — Number of homologs ranged from 1 (41 PDB IDS, accounting for a total of 465 mutants) to 29 (2PCC, 12 mutants). Some PDB IDs appear more than once, because not all calculations converged for all homolog structures. Note the particularly low RMSE for 1A22 and 1DAN, which dominate the statistics for number of homologs = 3 and 4. This may reflect a low error in the ΔΔGexperimental, due to quality experimental work in the Wells and Edgington labs, on proteins of medical interest. (DOCX) [file pone.0257614.s001.docx]

| Primary PDB ID | RMSE (kcal/mol) Using single structure | RMSE (kcal/mol) Using multiple structures | Number of homologs | Number of primary mutants |
| --- | --- | --- | --- | --- |
| 1A22 | 0.84 | 0.67 | 4 | 142 |
| 1A4Y | 1.49 | 1.49 | 1 | 45 |
| 1ACB | 2.13 | 2.13 | 1 | 6 |
| 1AHW | 1.23 | 1.23 | 1 | 10 |
| 1AK4 | 2.43 | 2.43 | 1 | 15 |
| 1CBW | 1.03 | 0.96 | 2 | 15 |
| 1CSE | 1.71 | 1.59 | 2 | 6 |
| 1DAN | 0.86 | 0.78 | 2 | 118 |
| 1DQJ | 2.23 | 2.21 | 10 | 34 |
| 1DVF | 2.07 | 2.07 | 1 | 35 |
| 1E96 | 1.15 | 1.15 | 1 | 6 |
| 1EAW | 0.86 | 0.86 | 1 | 27 |
| 1EMV | 1.18 | 1.24 | 13 | 51 |
| 1F47 | 0.87 | 0.87 | 1 | 12 |
| 1FC2 | 1.62 | 1.62 | 1 | 9 |
| 1FCC | 1.99 | 1.99 | 1 | 8 |
| 1FR2 | 3.17 | 3.17 | 1 | 1 |
| 1GC1 | 0.67 | 0.59 | 3 | 56 |
| 1HE8 | 1.01 | 1.01 | 1 | 10 |
| 1IAR | 1.26 | 0.97 | 3 | 36 |
| 1JCK | 1.52 | 1.52 | 1 | 7 |
| 1JRH | 1.62 | 1.62 | 1 | 53 |
| 1JTG | 2.73 | 2.29 | 5 | 118 |
| 1KAC | 1.90 | 1.45 | 2 | 2 |
| 1KIP | 0.63 | 0.63 | 1 | 1 |
| 1KIQ | 3.98 | 3.98 | 1 | 1 |
| 1KIR | 1.15 | 1.15 | 1 | 1 |
| 1KTZ | 1.15 | 1.15 | 1 | 27 |
| 1LFD | 0.85 | 0.85 | 1 | 25 |
| 1MLC | 0.97 | 1.01 | 2 | 11 |
| 1NCA | 0.84 | 0.86 | 4 | 4 |
| 1NMB | 2.12 | 1.86 | 3 | 6 |
| 1P69 | 0.42 | 0.42 | 1 | 1 |
| 1P6A | 3.76 | 3.76 | 1 | 1 |
| 1REW | 1.29 | 1.27 | 3 | 24 |
| 1S1Q | 1.02 | 1.02 | 1 | 6 |
| 1TM1 | 1.69 | 1.64 | 18 | 21 |
| 1UUZ | 1.09 | 1.09 | 1 | 5 |
| 1VFB | 1.15 | 1.03 | 9 | 42 |
| 1XD3 | 1.03 | 1.03 | 1 | 18 |
| 1Z7X | 1.33 | 1.36 | 2 | 21 |
| 2A9K | 0.92 | 0.92 | 2 | 1 |
| 2B42 | 0.69 | 0.69 | 1 | 3 |
| 2GYK | 4.67 | 4.67 | 2 | 1 |
| 2I26 | 0.59 | 0.59 | 1 | 2 |
| 2I9B | 0.47 | 0.47 | 4 | 5 |
| 2J12 | 0.95 | 0.95 | 1 | 1 |
| 2J1K | 1.09 | 1.09 | 1 | 3 |
| 2JEL | 1.44 | 1.44 | 1 | 43 |
| 2OOB | 0.72 | 0.72 | 1 | 4 |
| 2PCB | 1.17 | 0.90 | 3 | 6 |
| 2PCC | 1.76 | 2.03 | 17 | 12 |
| 2QJ9 | 1.37 | 0.79 | 2 | 3 |
| 2QJA | 1.06 | 1.19 | 2 | 3 |
| 2QJB | 1.40 | 1.40 | 1 | 3 |
| 2VLJ | 1.00 | 1.00 | 1 | 14 |
| 2VLN | 1.82 | 1.82 | 1 | 1 |
| 2VLO | 0.97 | 0.97 | 1 | 1 |
| 2VLP | 0.76 | 0.76 | 1 | 1 |
| 2VLQ | 1.03 | 1.03 | 1 | 1 |
| 2VLR | 0.19 | 0.19 | 1 | 1 |
| 2WPT | 1.85 | 1.85 | 1 | 32 |
| 3BK3 | 1.48 | 1.48 | 1 | 13 |
| 3BN9 | 0.65 | 0.65 | 1 | 25 |
| 3NPS | 0.88 | 0.88 | 1 | 27 |
