# Supplementary figures and images for: Mining the Protein Data Bank to improve prediction of changes in protein-protein binding
Source: PLoS One. 2021 Nov 2;16(11):e0257614. doi: 10.1371/journal.pone.0257614 (PMC8562805; doi:10.1371/journal.pone.0257614)

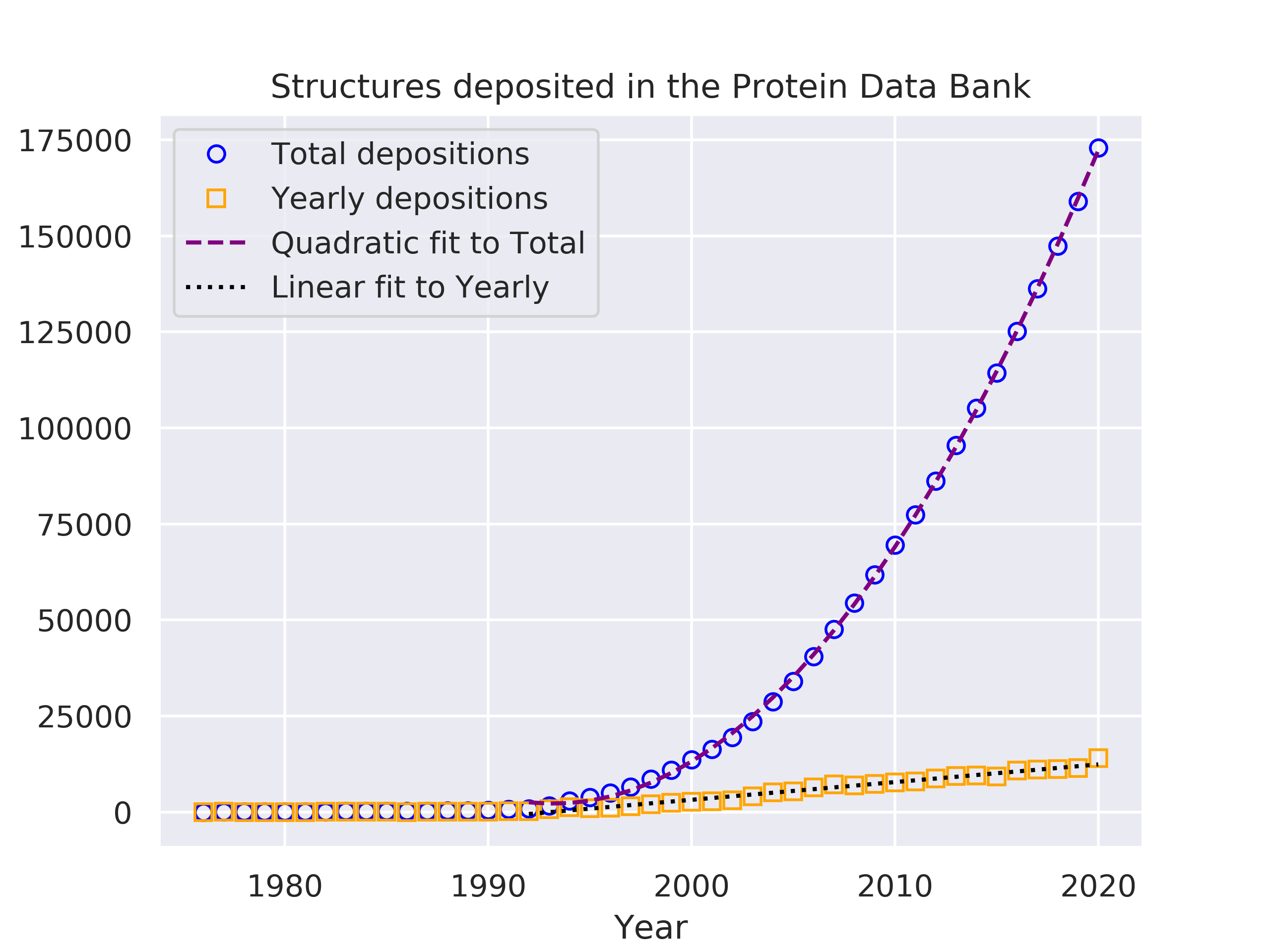

Supplement: S1 Fig — Depositions per year are growing approximately linearly (slope of 461 structures/year). Accordingly total structures are can be fitted to good approximation by 237*(year-1992)2–466*(year-1992) + 2523 (using numpy’s polyfit function). Interestingly, the largest increase was in 2020, despite (or perhaps because of) the Covid crisis [Acta Crystallogr D Struct Biol. 2020 Apr 1;76:311–312]. (TIF) [file pone.0257614.s002.tif]

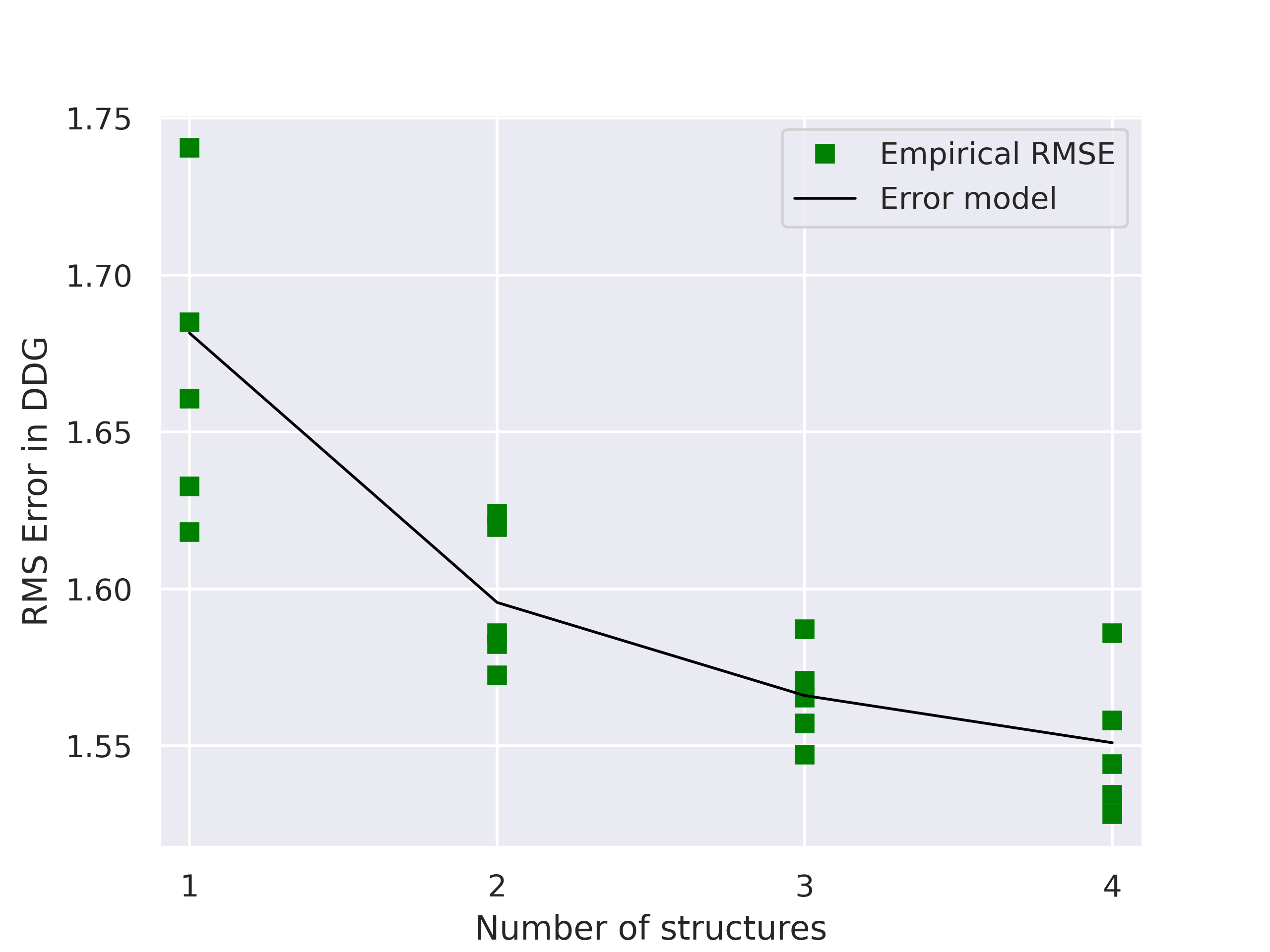

Supplement: S2 Fig — All data points are over the same set of mutants, namely single- and multiple-substitutions mutants, where 4 or more structures are available, N = 511. For all mutants, we randomly selected n = 1, 2, 3, and 4 of the available structures and computed RMSE; we repeated this five times. A maximum of n = 4 was selected to include the high-quality data associated with 1A22, and also to have a sufficiently high N. We suggest a model (based on normally distributed errors) in which total error σtotal is given by: σtotal=(σsinglestruct2n)+σsystematic2 Thus for large n, the total error would converge to σsystematic – the error due to the perturbative assumption, crystallization artifacts, biases in the underlying force field, etc. The RMSE qualitatively appears to have this convergence, though the data admit other models. (TIF) [file pone.0257614.s003.tif]
